# Birth Prevalence of Neural Tube Defects and Orofacial Clefts in India: A Systematic Review and Meta-Analysis

**DOI:** 10.1371/journal.pone.0118961

**Published:** 2015-03-13

**Authors:** Komal Preet Allagh, B. R. Shamanna, Gudlavalleti V. S. Murthy, Andy R. Ness, Pat Doyle, Sutapa B. Neogi, Hira B. Pant, Wellcome Trust- PHFI Folic Acid project team

**Affiliations:** 1 South Asia Centre for Disability Inclusive Development and Research, Indian Institute of Public Health-Hyderabad, Public Health Foundation of India, Hyderabad, India; 2 University of Hyderabad, Hyderabad, India; 3 National Institute for Health Research Bristol Nutrition Biomedical Research Unit at University Hospitals Bristol NHS Foundation Trust and the University of Bristol, Bristol, United Kingdom; 4 The UK National Institute for Health Research Bristol Nutrition Biomedical Research Unit in Nutrition, Diet and Lifestyle at University Hospitals Bristol NHS Foundation Trust and the University of Bristol and the School of Oral and Dental Sciences, University of Bristol, Bristol, United Kingdom; 5 London School of Hygiene and Tropical Medicine, London, United Kingdom; 6 Indian Institute of Public Health-Delhi, Public Health Foundation of India, Delhi, India; The Hospital for Sick Children, PAKISTAN

## Abstract

**Background:**

In the last two decades, India has witnessed a substantial decrease in infant mortality attributed to infectious disease and malnutrition. However, the mortality attributed to birth defects remains constant. Studies on the prevalence of birth defects such as neural tube defects and orofacial clefts in India have reported inconsistent results. Therefore, we conducted a systematic review of observational studies to document the birth prevalence of neural tube defects and orofacial clefts.

**Methods:**

A comprehensive literature search for observational studies was conducted in MEDLINE and EMBASE databases using key MeSH terms (neural tube defects OR cleft lip OR cleft palate AND Prevalence AND India). Two reviewers independently reviewed the retrieved studies, and studies satisfying the eligibility were included. The quality of included studies was assessed using selected criteria from STROBE statement.

**Results:**

The overall pooled birth prevalence (random effect) of neural tube defects in India is 4.5 per 1000 total births (95% CI 4.2 to 4.9). The overall pooled birth prevalence (random effect) of orofacial clefts is 1.3 per 1000 total births (95% CI 1.1 to 1.5). Subgroup analyses were performed by region, time period, consanguinity, and gender of newborn.

**Conclusion:**

The overall prevalence of neural tube defects from India is high compared to other regions of the world, while that of orofacial clefts is similar to other countries. The majority of studies included in the review were hospital based. The quality of these studies ranged from low to moderate. Further well-designed, high quality community-based observational studies are needed to accurately estimate the burden of neural tube defects and orofacial clefts in India.

## Introduction

The progress towards achieving the 4^th^ Millennium Development goal of reducing child mortality by two thirds by 2015 in India is slow, compared to other countries in the South East Asia Region [1]. There has been a decline in the number of deaths in children under five years of age in India from 2.5 million in 2001 to 1.5 million in 2012. Despite this decline India tops the list of countries with the largest number of under five deaths in the world [2].

There has been a decline in the number of deaths in infant and under-5 children attributed to infectious diseases and malnutrition in low and middle income countries; however the mortality attributed to birth defects remains constant [1]. Globally birth defects affect approximately 1 in 33 infants, resulting in an estimated 3.2 million children with birth defects every year [3]. The evidence suggests that the birth prevalence of all birth defects is 20% higher in low and middle income countries than in higher income countries [4]. In India, birth defects are listed as the cause of death in around 7% of deaths among under-5 children [5]. Birth defects are also reported to be the cause of 9.5% of perinatal deaths and 9.9% of still births in India [1].

The most common birth defect in India is neural tube defects (NTDs) [1]. The presentations of NTDs vary from anencephaly, encephalocoele to spina bifida occulta or cystica [6]. The risk of NTDs can be reduced by consumption of adequate amounts of folic acid prior to conception and in early pregnancy [1]. Orofacial clefts (OFCs) are another set of common birth defects in India, the prevalence of which has been suggested to be reduced by peri-conceptional intake of folic acid [7]. Orofacial clefts are broadly divided into cleft lip with or without cleft palate and cleft palate only [8].

India is one of the many regions of the world where population estimates of the prevalence of birth defects are not routinely collected [9]. Currently there is no national registry for birth defects. Hospital based surveys or studies are the most common source of information on birth defects like NTDs and OFCs in India.

In India, several studies have reported varying results on the prevalence of NTDs and OFCs. This may be a result of geographical variation, the different criteria used in data collection, the case definition used and other methodological issues like variation in quality of the study design [4].

The aim of the current review is to determine the prevalence of neural tube defects and orofacial clefts among live births and still births in India with all available community and hospital based observational studies.

A systematic review on the birth prevalence of neural tube defects in India has been reported earlier [10]. It reports a birth prevalence of NTDs as 4.1 cases per 1000 total births (95% CI 3.1–5.4 per 1000 total births). However in our review we have included larger studies from across India (547,803 new-borns) compared to the earlier review (308,387 new-borns). In addition, we have conducted a sub-group analysis on relation of time, gender of newborn, region and consanguinity on the prevalence of NTDs. We also report a systematic review on birth prevalence of orofacial clefts in India.

## Methods

### Search Strategy

We performed a literature search on MEDLINE and EMBASE for articles using the following MeSH (Medical Education Subject Headings) terms: neural tube defects, cleft lip, cleft palate, India, prevalence; published up to 19th February 2013. For example on MEDLINE, we used the following search strategy “((((neural tube defects) OR cleft lip) OR cleft palate) AND India) AND Prevalence”. To optimize our search, hand searches of reference lists of included articles were also performed.

### Study Selection

Two authors (KPA and BRS) independently assessed titles and abstracts for eligibility, and any disagreement was resolved through discussion. We obtained a copy of the full text for all papers that were available and included. The included studies are depicted in Table 1.

**Table 1:** Summary of studies included in the review; the studies in the table are presented based on the region of the country where the study was conducted.

Place of study	Year of publication	Setting	Study type	Study duration	Total no. of babies in study (denominator)	Age of children	Prevalence of NTD/1000 births	Prevalence of OFCs/ 1000 births
**NORTH INDIA**								
Delhi [11]	1991	Hospital	Cross-sectional survey	January 1988 to August 1990	9220	At birth*	7.1	-
Ludhiana[12]	1991	Hospital	Review of hospital records	January 1983 to March 1989	10000	At birth*	4.7	1.4
Rohtak [13]	1992	Hospital	Cross-sectional survey	June to September 1989	4785	At birth	18.2	-
Varanasi[14]	1994	Hospital	Cross-sectional survey	January 1988 to December 1999	3932	At birth*	2.5	0.3
Delhi [15]	1998	Hospital	Cross-sectional survey	Over a period of three years	23367	At birth*	7.9	-
Shimla [16]	2000	Hospital	Cross sectional survey	January 1991 to December 1995	10100	At birth*	4.5	-
Balrampur [17]	2005	Community	Review of births in community in women who delivered at the hospital in Utraula	October 2002 and September 2003	1218	0–1 years	8.2	-
**SOUTH INDIA**								
Mysore [18]	1970	Hospital	Review of hospital records	1967 to 1969	5554	At birth*	1.3	0.2
Davangere [19]	1987	Hospital	Cross-sectional survey	March- Aug 1984–1987	5500	At birth*	11.3	-
Pondicherry [20]	1998	Hospital	Cross-sectional survey	September 1989 to December 1992	12797	At birth*	3.2	2.0
Pondicherry [21]	2005	Hospital	Cross-sectional survey	July 1998- June 2004	54738	At birth*	5.7	-
Kollam [22]	2013	Hospital	Cross-sectional survey	August 1995 to June 2011	141540	At birth*	0.5	1.1
**WEST INDIA**								
Bombay [23]	1968	Hospital	Cross-sectional survey	1960 to 1963	23568	At birth*	2.1	-
Wardha [24]	1989	Hospital	Cross-sectional survey	April 1985 to March1986	3014	At birth*	4.0	2.4
Wardha [25]	2000	Hospital	Cross sectional survey	April 1998 to April 1999	2968	At birth*	1.7	1.0
Delhi/ Mumbai/ Baroda [26]	2002	Hospital	Cross sectional survey		94610	At birth*	3.6	0.9
Wardha [27]	2010	Hospital	Cross sectional survey	January 2005 to July 2007	9386	At birth*	0.7	1.0
Ahmedabad [28]	2012	Hospital	Cross sectional survey	Not mentioned	5240	At birth*	4.2	2.9
**EAST INDIA**								
Calcutta [29]	1989	Hospital	Review of hospital records of 5 hospitals Cross sectional survey	1976 to 1987 1986 to 1987	126266	At birth (live births)	1.1	0.7

* Live births and still births

The studies had to fulfil the following criteria to be eligible for inclusion in the review:

1. Study setting—community or hospital-based and have a clearly defined target population;
2. Type of participants- Live births and still births;
3. Type of outcome- neural tube defects (Anencephaly/ Encephalocoele/ Iniencephaly/ Spina Bifida/ Craniorachischisis/ Hydranencephaly); orofacial clefts (cleft lip/ cleft palate/ cleft lip with cleft palate);

Exclusion criteria were as follows:

1. Case reports and case series;
2. Studies where OFCs and NTDs were not clearly defined and reported separately
3. Studies focusing on treatment of congenital malformations.

### Data Extraction

We designed a data extraction form in Excel to extract relevant data for our review. For each study that fulfilled the criteria, we extracted the following information: first authors name, year of publication, study setting (hospital or community), study design, duration of study, geographical setting, participant age, gender of newborn, history of consanguinity, total sample size, type of neural tube defect or orofacial cleft, prevalence per 1000 births of the outcome (NTDs or OFCs)

For included studies, two authors (KPA and BRS) extracted the data using the agreed form. We entered data into Review Manager Software (version 5.2) and carried out checks for accuracy.

### Data Analysis and Statistical Methods

Birth prevalence was calculated as total number of new-born’s affected with NTDs or OFCs per 1000 total births (live and still births). In studies where the Standard Error (SE) was not reported, we calculated it from the prevalence using the following formula: SE = √p (1-p)/ n & 95% CI = p ± 1.96 X SE; where, p = Prevalence.

Meta-analysis was performed using Review Manager software (Version 5.2). The heterogeneity of each meta-analysis was assessed and then both the random effects and fixed effects model was used to calculate the pooled prevalence. This helped in comparing the estimates that each produced. We conducted subgroup analysis using the following: regions of India (North/South/ East/ West); gender; history of consanguinity and studies reporting data before, versus after 1995. Funnel plots were used to study the possibility of publication bias.

### Quality Assessment

In order to determine the quality of the included studies we used six criteria based on the STROBE statement [30] to grade the included studies, as seen in Table 2. The criteria include (i) clear description of the study setting; (ii) clear description of the study population; (iii) details on how the diagnosis of NTDs or OFCs was made; (iv) whether informed consent was taken to be a part of the study; (v) whether the study examined consecutive births; and (vi) whether the results of the study can be generalised to the wider community. Two authors assessed the quality of the included studies. A criteria was marked a ‘+’ sign if it was fulfilled by the study. A study was considered of poor quality if it did not meet more than 2 criteria. Any disagreements between reviewers were resolved by discussion.

**Table 2:** Quality Assessment of Individual studies based on the STROBE criteria.

	Description of study setting	Description of study population	How diagnosis made	Information on consent taken	Consecutive births examined	Results of study generalised
Ahmedabad, 2012	-	+	+	+	-	-
Balrampur, 2005	+	+	+	+	+	+
Bombay, 1968	+	+	-	-	+	-
Calcutta, 1989	+	+	-	-	+	+
Davangere, 1987	+	+	-	-	+	-
Delhi, 1991	+	+	+	-	+	-
Delhi, 1998	+	+	+	-	+	-
Delhi, Mumbai, Baroda, 2002	-	+	-	-	+	-
Kollam, 2013	+	+	+	-	+	+
Ludhiana, 1991	+	+	+	-	+	+
Mysore, 1970	+	+	-	-	+	-
Pondicherry, 1998	+	+	+	-	+	-
Pondicherry, 2005	+	+	+	-	+	+
Rothak, 1992	+	+	**+**	-	+	-
Shimla, 2000	+	+	+	-	+	+
Tamil Nadu, 2009	+	+	+	+	-	+
Varanasi, 1994	+	+	+	-	+	+
Wardha, 1989	+	+	+	-	+	-
Wardha, 2000	+	+	+	-	+	-
Wardha, 2010	+	+	+	-	+	-

‘+’ the study meets the criteria;

‘-’ the study does not meet the criteria

## Results

### Studies included

**Fig 1:**
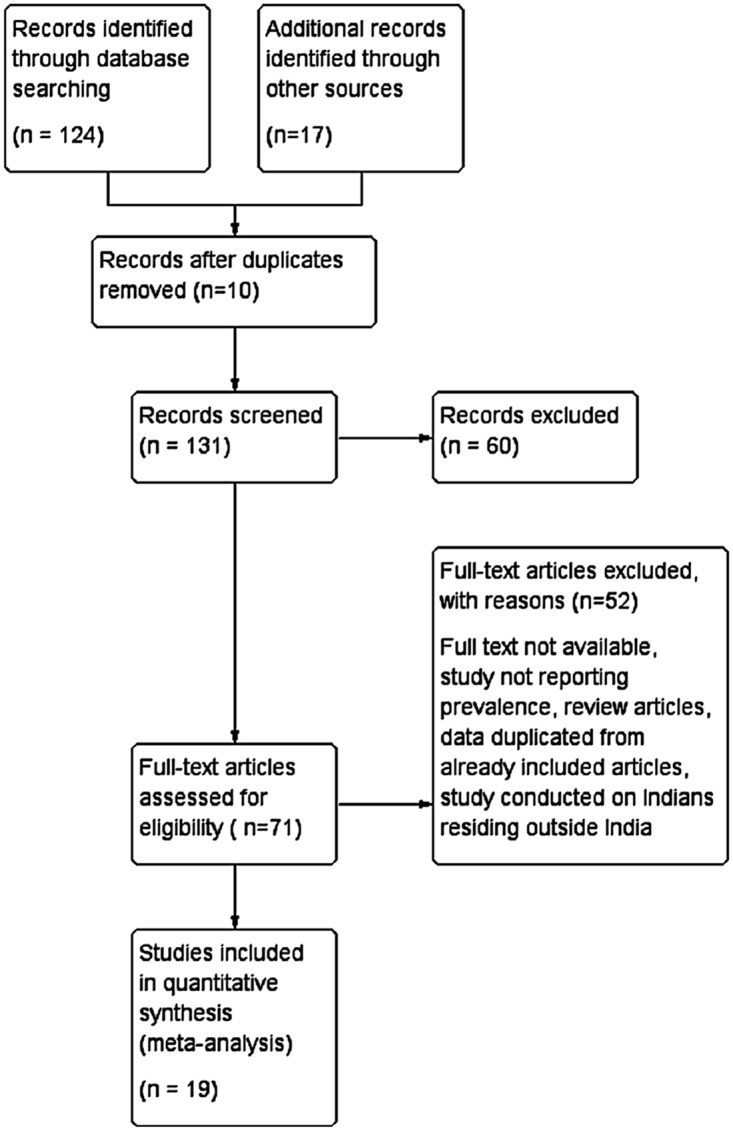
Literature search and selection of studies (PRISMA Flow diagram).

A total of 131 articles were identified from the search strategy (114 articles) and hand searches of reference lists (17 articles) of included articles were also performed. From these, 112 articles were excluded. Nineteen articles met our inclusion criteria. The articles reported the birth prevalence of neural tube defects and/or orofacial defects. Fig. 1 depicts the Prisma flow diagram.

### Study characteristics

Table 1 presents the characteristics of the individual included studies. The studies which were included were from 1968 upto 2013. The sample sizes of the included studies ranged from 1,218 births to 141,540 births. Except for one community based study, all included studies were hospital based. We have studies representing most regions of India; however there was just one study from the eastern region of India and no studies from the central and north east region of India.

### Prevalence of neural tube defects in India

**Fig 2:**
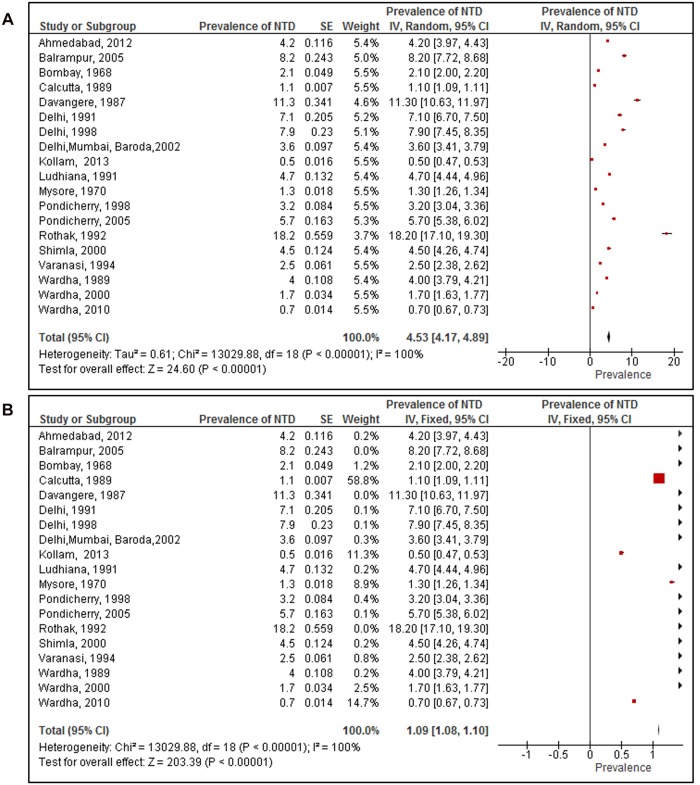
Prevalence of neural tube defects among studies included in the systematic review and meta-analysis. (A) Pooled prevalence of neural tube defects using the random effects model. (B) Pooled prevalence of neural tube defects using fixed effects model.

Nineteen studies that reported on prevalence of NTD among 547,803 new-borns were included. The birth prevalence of NTDs reported ranged from 0.5 per 1000 total births [22] to 18.2 per 1000 total births [13]. All the studies included in the meta-analysis were hospital based studies except one. This was a community based door-to-door survey carried out in the Balrampur district of Uttar Pradesh, India [17]. The results of meta-analysis (random effects model; Fig. 2A) showed the overall pooled prevalence of NTDs in India was 4.5 per 1000 total births (95% CI 4.2 to 4.9). This was calculated from the 19 studies included in the meta-analysis. The heterogeneity was high (I^2^ = 100%).

By contrast, the fixed effect model (Fig. 2B) showed the overall pooled prevalence of NTDs in India was 1.09 per 1000 total births (95% CI 1.09 to 1.10). It was possible that the prevalence was weighted down by the contribution of a large study from Calcutta [29], which reported a low prevalence of NTD of 1.05 per 1000 births. This study had a particularly large sample size (of 126,266), and contributed 59% of the weight. However, upon recalculation of the pooled prevalence after removing this study, the prevalence was still 1.08 (95% CI 1.06 to 1.10).

The pooled estimates (random effects) for birth prevalence of NTDs among consanguineous marriages was 11.5 per 1000 total births (95% CI 2.5 to 20.5) versus 4.3 per 1000 total births (95% CI 0.5 to 8.1) in non-consanguineous marriages. There was no statistical evidence to show that children of consanguineous marriages have a higher prevalence of NTD.

Results from two studies [21, 22] with gender specific prevalence of NTDs, shows the pooled prevalence of 2.3 cases per 1000 male births (95% CI 0.45 to 5.14) and 4.3 cases per 1000 female births (95% CI 1.90 to 10.41). There was no statistical evidence that NTD prevalence was higher in any gender.

We compared the prevalence of NTDs in studies that collected data before 1995(9 studies) and after 1995 (10 studies). We choose 1995 as the cut-off point because half of the included studies were conducted before 1995 and the other half after 1995. The results from the meta- analysis showed that the pooled prevalence (random effects) of NTDs prior to 1995 was 5.3 per 1000 total births (95% CI 4.7 to 5.9) and after 1995, it was 4.0 per 1000 total births (95% CI 3.3 to 4.7). There was strong statistical evidence that the prevalence before 1995 was higher that than reported after 1995 (p = 0.004).

### Regional differences in prevalence of NTDs in India

We conducted a subgroup analysis to estimate regional differences in prevalence of NTDs. We had studies from four regions of India: northern, southern, western and eastern regions. [31]. The northern region of India includes the six states of Jammu and Kashmir, Himachal Pradesh, Haryana, Punjab, Uttarakhand and Uttar Pradesh. The four states of South India are Andhra Pradesh, Karnataka, Kerala and Tamil Nadu. The Western region covers the States of Rajasthan, Gujarat, Maharashtra and Goa. And the four states of East India are West Bengal, Bihar, Jharkhand and Odisha.

The highest pooled prevalence of NTDs was from the Northern region (7.7 per 1000 total births; 95% CI 5.5 to 9.6). The lowest birth prevalence of NTDs was reported from the eastern region (1.1 per 1000 total births; 95% CI 1.09 to 1.11). It should be noted that there was just one study reporting prevalence from the Eastern region of India. The Western region reported a pooled prevalence of 2.5 per 1000 total births (95% CI 1.6 to 3.5), while the Southern region reported 4.2 per 1000 total births (95% CI 3.4 to 5.1). There was strong statistical evidence that there were differences between regions, p <0.00001. This suggests that in Southern India, certain practices like consanguineous marriages, dietary factors, delayed age at marriage and child birth could lead to a higher prevalence of NTDs.

Visual inspection of the funnel plot showed that there was asymmetry which shows possibility of publication bias. Table 3 is a summary of the data for studies with NTDs; it shows comparison of results obtained by both random and fixed effect model. There was a significant difference in subgroup analysis of consanguinity: whereas in the fixed effect model, consanguineous marriages resulted in higher prevalence of NTDs, there was no similar statistical evidence by the random effect model.

**Table 3:** Summary table of the data from studies included on neural tube defects; the table depicts comparative data using fixed and random effects model.

Sub group	Sample size (n)	No of studies included	Random effect model	P value	Fixed effect model	P value
**Overall prevalence**	547,803	19	4.53 (4.17 to 4.89)		1.09 (1.08 to 1.10)	
**Region wise prevalence**		18				
North	62,622	7	7.56 (5.49 to 9.62)	P<0.00001*	3.86 (3.77 to 3.95)	P<0.00001*
South	220,129	5	4.24 (3.36 to 5.12)		0.94 (0.91 to 0.96)	
East	126,266	1	1.10 (1.09 to 1.11)		1.10 (1.09 to 1.11)	
West	44,176	5	2.53 (1.57 to 3.50)		1.0 (0.98 to 1.03)	
**Consanguinity**	201,778	3				
History of consanguinity			11.5 (2.48 to 20.52)	**P = 0.15**	2.23 (2.14 to 2.31)	**P<0.00001** ^*^
No history of consanguinity			4.29 (0.50 to 8.07)		0.58 (0.55 to 0.61)	
**Time trends**		19				
Before 1995	191,839	9	5.29 (4.71 to 5.86)	P = 0.004*	1.19 (1.18 to 1.20)	P<0.00001*
After 1995	340,624	10	3.98 (3.30 to 4.65)		0.87 (0.85 to 0.89)	
**Gender**	196,278	2				
Male			2.34 (0.45 to 5.14)	P = 0.58	1.11 (1.06 to 1.16)	P = 0.26
Female			4.26 (1.90 to 10.41)		1.14 (1.12 to 1.16)	

*Statistically significant

### Prevalence of orofacial clefts in India

**Fig 3:**
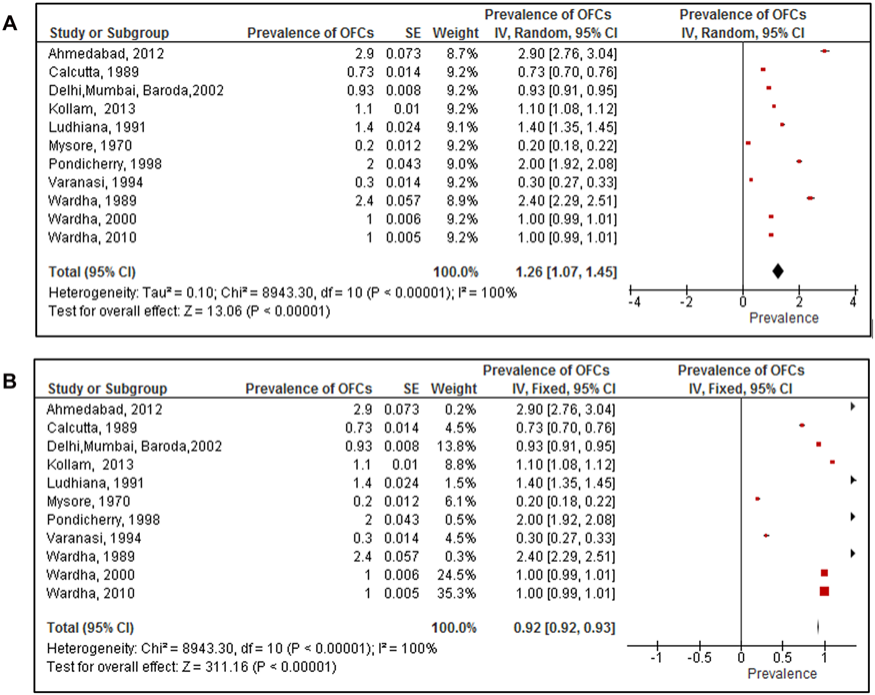
Prevalence of orofacial clefts among studies included in the systematic review and meta-analysis. (A) Pooled prevalence of orofacial clefts using the random effects model. (B) Pooled prevalence of orofacial clefts using fixed effects model.

Eleven studies that reported on the prevalence of OFCs in 415,307 new-borns were included. The reported prevalence of OFCs ranged from 0.2 per 1000 total births [18] to 2.9 per 1000 total births [28]. All studies included in the meta- analysis were hospital-based. The results from the meta-analysis (random effects; Fig. 3A) showed the overall pooled prevalence of OFCs in India was 1.3 per 1000 total births (95% CI 1.1 to 1.5). This was calculated from 11 studies included in the review. The heterogeneity was high (I^2^ = 100%). Despite this heterogeneity the results of meta-analysis (fixed effect; Fig. 3B) produced a similar estimate of the pooled prevalence of OFCs: 0.92 per 1000 total births (95% CI 0.92 to 0.93).

We compared the prevalence of OFCs in studies that collected data before 1995(5 studies) and after 1995 (6 studies). We choose 1995 as the cut-off point because half of the included studies were conducted before 1995 and the other half after 1995. The results from meta- analysis showed that the pooled prevalence (random effects) of OFCs prior to 1995 was 1 per 1000 total births (95% CI 0.6 to 1.4); and after 1995 it was 1.4 per 1000 total births (95% CI 1.3 to 1.6). There was no statistical evidence that the prevalence before 1995 was higher or lower that than reported after 1995 (p = 0.07).

There were two studies [22, 29] which reported gender specific prevalence of the OFCs in India. The pooled prevalence of OFCs among male births was 0.6 per 1000 total births and 0.53 per 1000 among female total births. There was no statistical evidence to prove any difference in prevalence of OFCs based on gender of the child.

### Regional differences in prevalence of OFCs in India

Similarly, we carried out a sub group analysis for prevalence of OFCs across the regions of India. The pooled prevalence in the Southern region [18, 20, 22] was 1.1 per 1000 total births (95% CI 0.3 to 1.9), and in the Western region [24, 25, 27] it was 1.8 per 1000 total births (95% CI 1.5 to 2.1). In the Eastern region [29] there was only one study reporting the prevalence of OFCs which was 0.70 per 1000 total births (95% CI 0.67 to 0.73). In the northern region the prevalence was 0.9 per 1000 births (95% CI 0.2 to 1.9). There was strong statistical evidence that the prevalence of OFCs was higher in the Western region compared to other regions and lowest in the eastern region of India (p<0.00001). This suggests that certain dietary and cultural practices in the western states of India could be the reason for higher prevalence of OFCs.

Visual inspection of funnel plot showed asymmetry, which indicates possibility of publication bias. Table 4 is a summary of the data for studies with OFCs; comparing results obtained by both random and fixed effect model. There is significant difference in subgroup analysis of gender and time trend; wherein by the fixed effect model, there is evidence of increase in prevalence of OFCs after 1995, but there was no such evidence by random effect model. Also the fixed effect model shows that prevalence of OFCs is higher in females than in males, which could not be proved by the random effects model.

**Table 4:** Summary table of the data from studies included on orofacial clefts; the table depicts comparative data using fixed and random effects model.

Sub group	Sample size (n)	No of studies included	Random effect model	P value	Fixed effect model	P value
**Overall prevalence**	410,067	11	1.26 (1.07 to 1.45)		0.92 (0.92 to. 93)	
**Region wise prevalence**		10				
North	13,932	2	0.85 (0.23 to 1.93)	P <0.00001*	0.87 (0.86 to 0.88)	P<0.00001*
South	159,891	3	1.10 (0.33 to 1.87)		0.77 (0.76 to 0.79)	
East	126,266	1	0.70 (0.67 to 0.73)		0.70 (0.67 to 0.73)	
West	15,368	4	1.78 (1.5 to 2.05)		0.99 (0.98 to 1)	
**Time trends**		11				
Before 1995	148,766	5	1 (0.55 to 1.44)	**P = 0.07**	0.5 (0.49 to 0.52)	**P<0.00001** ^*^
After 1995	261,301	6	1.43 (1.31 to 1.55)		1 (1 to 1.01)	
**Gender**	267,806	2				
Male			0.60 (0.02 to 1.18)	**P = 0.87**	0.71 (0.70 to 0.73)	**P<0.00001** ^*^
Female			0.53 (0.02 to 1.09)		0.81 (0.80 to 0.82)	

* Statistically significant

## Discussion

The prevalence of neural tube defects and orofacial clefts varied widely across studies. We used meta-analysis to combine the findings of studies we identified by a systematic review of the published literature. For the meta-analysis of NTDs, we included nineteen studies. We obtained a pooled birth prevalence of 4.5 per 1000 total births using the random effect model. This estimated prevalence in our review was high compared to other regions of the world. For instance in the United States, the prevalence of NTDs is 0.7 cases per 1000 births; Canada it is 0.7 cases per 1000 births; Chile it is 0.9 per 1000 births; and in South Africa it is 1 per 1000 births [32]. However the birth prevalence is lower in comparison to our neighbouring country Pakistan, where the birth prevalence is 13.8 cases per 1000 births [33].

In the meta-analysis of orofacial clefts, eleven studies were included. The pooled birth prevalence was 1.3 cases per 1000 total births. This prevalence was similar to that in other regions of the world. For example, in Africa, the birth prevalence of OFCs ranges from 0.3 cases per 1000 births in Nigeria; to 1.7 cases per 1000 births [7]. In the United States, prevalence at birth ranges from 0.5 to 1.7 cases per 1000 live births [34]. In Ireland, a recent study reported a prevalence of 1.6 per 1000 births [35].

The results of prevalence for NTDs using random versus fixed effect model were very different (4.5 cases vs. 1.1 cases per 1000 births). But these results were similar for prevalence of OFCs (1.3 cases vs. 0.9 per 1000 births).

The results from our meta-analysis, shows a pooled prevalence of NTDs similar to pooled prevalence (4.1 per 1000 births) projected by Bhide et al. [10]. We have presented and compared results using both fixed and random effects model. However, Bhide et al presented results only using the random effect model. On plotting the funnel plot, there was a difference in results obtained from Bhide et al and our review. We found funnel plot asymmetry, suggestive of publication bias which was not observed by Bhide et al. This asymmetry can be attributed to reporting biases like publication bias, language bias, citation bias, etc.; different methodological quality of the included studies, or the asymmetry could occur due to chance, when few numbers of studies are included.

Results from this meta-analysis suggest that the birth prevalence of both NTDs and OFCs varies across the different regions of India. As depicted in Table 3, there was a seven fold difference in the prevalence of NTDs across regions of India. The prevalence at birth was highest in the Northern region at 7.7 cases per 1000 births, while prevalence in the Eastern region was 1.1 cases per 1000 births. Such variation could be explained by lower compliance with folic acid supplementation among women in North India as reported by District level household survey -3 [36]; or by variation in diets [37] and various other socio cultural factors that may be attributable to the outcomes beliefs. From our meta-analysis, there is no evidence to show gender based difference in prevalence of NTDs. In children born to consanguineous parents, the prevalence of NTDs is 11.5/1000 total births, whereas in children of non-consanguineous parents, the prevalence of NTDs is 4.3/1000 total births. However the p value (p = 0.15) does not show statistical evidence that there is a significant difference.

For orofacial clefts there was a twofold difference in birth prevalence across regions. The prevalence of OFCs was highest in West India with 1.8 cases per 1000 births and lowest in the East India with 0.7 cases per 1000 births. The prevalence of NTDs and OFCs was highest in different regions of India, suggesting the underlying aetiology of the two conditions may be different.

The review shows a considerable reduction in prevalence estimates of NTDs after 1995. Prior to 1995 there are 5.3 cases per 1000 births and 4 cases per 1000 births after 1995. This reduction in birth prevalence could be attributed to increased awareness of the importance of folic acid supplements during pregnancy, given the fact that most of these are hospital based studies from big cities. Another possibility could be early detection of NTDs during pregnancy followed by abortion. No such differences were noted in the birth prevalence of OFCs.

These prevalence estimates in our review may be an over representation as majority of the included studies were hospital-based. And in determining the true population prevalence of NTDs and OFCs, the source of data whether hospital or community based; has a major bearing on the results. For instance, if the source of data is from hospitals, there is a possibility that due to referral of cases with complex birth defects, there could be an overestimation of prevalence of NTDs in hospitals. On the other hand, from a community setting, deliveries of anencephaly cases may lead to still births or missed abortions. These cases may never reach a hospital and thus deliveries of such still births in the community can lead to underestimation of prevalence of birth defects from hospital based data.

In our review, among the included studies there was just one community based study from Uttar Pradesh (Northern region, India) which reported a prevalence of NTDs as 8.2 cases per 1000 live births [17]. However, this study reports the possibility of underestimation of prevalence because data on still births was not included. Another reason for underestimation would be that the community may not report a case due to social taboos. A community based prevalence study of OFCs in a Tamil Nadu (Southern region, India) among 11.8 million children (0 to 15 years) reported a prevalence of OFCs of 4.7 cases per 10,000 live children [38]. However, this study was not included in the review as it did not meet the inclusion criteria.

Another factor that would affect the prevalence of individual studies is whether the women included in the study have a past history of NTDs. The risk of recurrence of NTD after birth of one affected child is 3–5%, which is 10 times higher than that of the general population [39]. This would lead to an increased overall prevalence, if the study included two cases of NTD from the same mother in the study period. However, among all the studies included in the meta-analysis, none have included the two cases of NTDs from same mother in the study period.

### Strengths and limitations of Systematic review

The strengths of this review are that we systematically identified and included prevalence estimates from 1963 onwards for NTDs and OFCs [23]. We have used meta-analysis to derive a pooled prevalence estimate of all the included observational studies. We carried out a quality assessment of the included studies based on criteria from the STROBE statement.

However this review has a few limitations. We have only conducted the search in electronic databases. Studies published in local journals which are not indexed in PubMed might have been missed out in this review. We did not include non- English language published studies in the analysis. In the community based studies there may have been under reporting of cases of both NTDs and OFCs due to the mother and the family withholding information due to cultural norms or feeling of shame at having a baby with a birth defect. Another limitation was that our MeSH terms were limited to NTDs broadly, but specific diseases like spina bifida, anencephaly, and encephalocoele were not included.

### Policy implications and recommendations for future research

Despite a reduction in the prevalence of NTDs since 1995 the overall prevalence of NTDs is still high. There is a seven fold variation between regions of India. This suggests that preventative policies aimed at reducing NTDs need to be strengthened.

Further descriptive studies to explain regional differences in the birth prevalence of NTD are required. Most of the current studies that report the prevalence are hospital based studies and may underestimate community prevalence of these NTDs and OFCs because many such children milder and subtler ones, do not report to the hospitals unless until they are captured in a well-designed and dynamic registry system. It should also be understood that children with NTDs tend to die earlier and hence in cases of serious birth defects they may be underestimated. Large, ongoing high-quality community based studies are required to monitor the prevalence of NTDs and OFCs in India. This would help in developing and strengthening current policies in India on prevention of NTDs and OFCs.

Lastly, there is a need to set up a National level registry in India, where all cases of any birth defect are documented. The registry will have a large sample size which can help evaluate potential risk factors for specific birth defects. Also it can help to accurately determine the regional differences of birth defects across the country, which can help guide future research. The Government of India has initiated a new scheme ‘Rashtriya Bal Swasthya Karyakram’ (RBSK) [40], aiming at early identification and early intervention from birth to 18 years to cover defects at birth including NTDs and OFCs. This information can provide country wide epidemiological data on birth defects. However this initiative is still in the nascent stage.

### Conclusions

The prevalence of NTDs at birth is high in India, compared to other regions of the world. While the prevalence of OFCs at birth, is similar to that in other parts of world. The prevalence of NTDs varies over time and regions of India suggesting that this condition is preventable. The present data on birth prevalence of NTDs and OFCs is of poor quality and hence there is a need to conduct better studies to monitor the burden of disease related to NTD.

## Supporting Information

S1 PRISMA Checklist (DOC)
